# Peroneal Nerve Palsy due to Bulky Osteochondroma from the Fibular Head: A Rare Case and Literature Review

**DOI:** 10.1155/2020/8825708

**Published:** 2020-11-12

**Authors:** T. Cherrad, M. Bennani, H. Zejjari, J. Louaste, L. Amhajji

**Affiliations:** ^1^Department of Orthopedic Surgery and Traumatology, Military Hospital Moulay Ismail Meknes (HMMI), Morocco BP 50000; ^2^Faculté de médecine et pharmacie de Fès, CHU HASSAN II Fès, Université Sidi Mohamed Ben Abdelah, Fés, Morocco

## Abstract

Common peroneal neuropathy is the most common compressive neuropathy in the lower extremities. The anatomical relationship of the fibular head with the peroneal nerve explains entrapment in this location. We report the case of a 14-year-old boy admitted with a left foot drop. The diagnosis was an osteochondroma of the proximal fibula compressing the common peroneal nerve. The patient underwent surgical decompression of the nerve and resection of the exostosis. Three months postoperatively, there was a complete recovery of the deficits. The association of osteochondroma and peroneal nerve palsy is rare. Early diagnosis is required in order to adjust the management and improve the results. It is worth to underscore that surgical resection is proven to be the appropriate treatment method ensuring high success rates.

## 1. Introduction

Common peroneal nerve (CPN) palsy related to compression is the most frequent nerve entrapment in the lower extremities and the third most common entrapment in the body after the median and ulnar nerve entrapment [1, 2]. The predisposition of the CPN to such entrapment is thought to be due to the anatomical course: at the fibular head, the nerve is superficial and covered only by subcutaneous tissue and fat; it wraps around the neck of the fibula and runs under arching peroneus longus muscle fibers. This osteofibrous tunnel is the site of a potential entrapment of the nerve. Mechanical entrapment by an osteochondroma is seldom [3].

A case of nerve compression caused by a benign osteophyte-like lesion is presented. The mechanism, the diagnostic approach, and the treatment strategy are highlighted.

## 2. Case Report

A 14-year-old boy was seen in our department as an outpatient with complaints of difficulty in walking, gradual weakness of foot dorsiflexion, and tingling and numbness of the back of the left foot. The appearance of these symptoms dated back to 3 months prior to his admission which were manifested following a direct trauma of the left knee's lateral side that happened during a soccer match. Moreover, during this period, the patient reported the presence of a painless mass on the lateral side of the left knee with a gradual increase of its volume.

Clinical examination revealed limping and a hard lump over the left fibular head. His neurological examination showed paralysis of his tibialis anterior (0/5), extensor hallucis longus (0/5), extensor digitorum longus and brevis (0/5), and peroneal muscles (1/5). The Tinel sign was present with a sensory deficit at the anterolateral region of the lower leg and dorsum of the foot. Nerve conduction study (NCS) and electromyography (EMG) of left CPN showed normal distal latency (DL) and normal compound action potential (CMAP) as the nerve was stimulated at the ankle, but during CPN stimulation at the knee, there were prolonged DL and severely decreased CMAP (>50%) in comparison to distal stimulation that indicated nerve conduction block with temporal dispersion. EMG of the muscles supplied by the CPN was normal.

The rest of the examination did not reveal frontal and sagittal laxity of the knee, nor other abnormalities regarding the musculoskeletal and neurological system.

**Figure 1:**
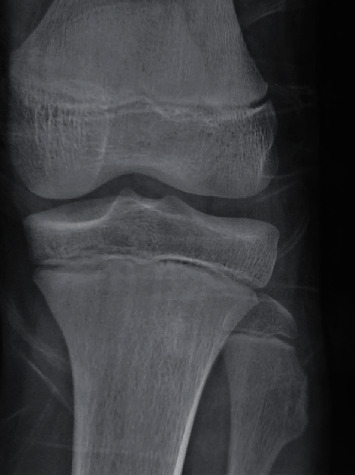
Anteroposterior plain films of the left knee showing an osteophytic protuberance in the neck of the fibula.

X-ray and ultrasound detected compression of the peroneal nerve due to the osteophyte protuberance located at the neck of the fibula (Figure 1).

**Figure 2:**
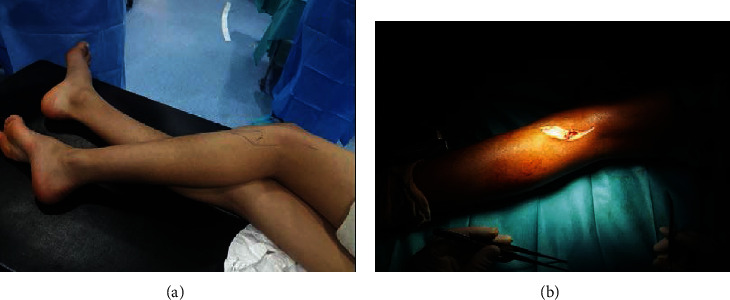
(a) The patient in lateral decubitus position on the operating table. (b) The posterolateral approach of the knee with an incision in a hockey stick shape.

**Figure 3:**
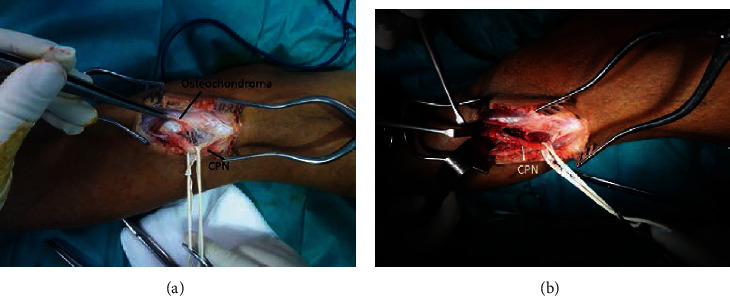
(a) Peroneal nerve looks flattened and inflamed due to osteochondroma at the level of fibular neck. (b) Intraoperative picture showing the CPN after removal of the osteochondroma.

The patient underwent surgical decompression of the left peroneal nerve. A large exostosis of the fibular head, compressing and displacing the CPN, was observed. The bone lesion was removed, and careful neurolysis was performed (Figures 2 and 3). The histopathologic study confirmed the diagnosis of osteochondroma.

**Figure 4:**
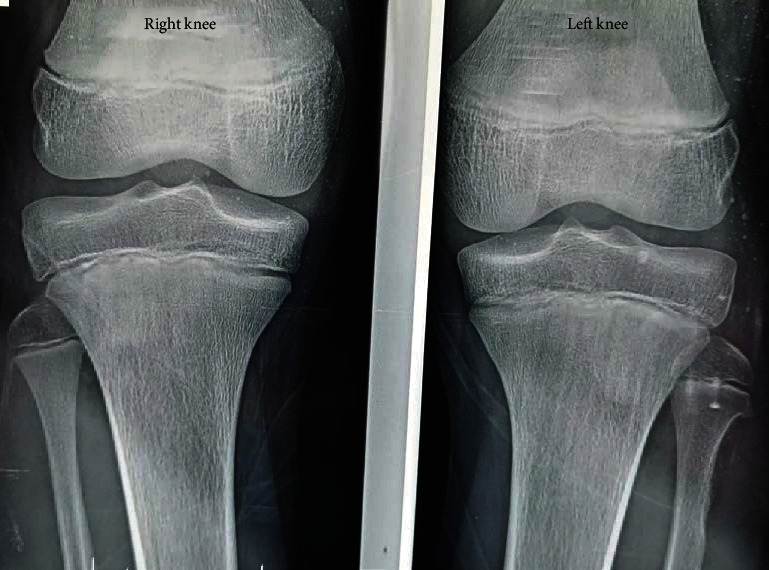
Plain X-rays of both knees directly postoperatively.

**Figure 5:**
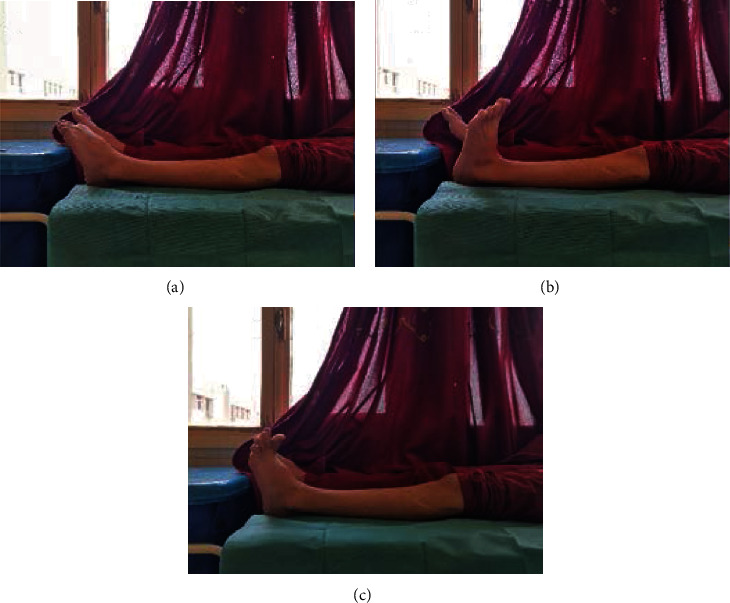
Full recovery of the preoperative deficit after three months: (a) foot flexion, (b) foot extension, and (c) hallux extension.

Postoperatively, the patient was discharged with a foot drop polyethylene splint and physiotherapy sessions. Progressive clinical improvement was observed; three months postoperatively, the deficit was recovered (Figures 4 and 5).

## 3. Discussion

The CPN is the lateral branch of the sciatic nerve. It travels inferiorly and laterally from its origin at the apex of the popliteal fossa through the posterior compartment of the thigh along the medial border of the biceps femoris tendon and posterior to the lateral head of the gastrocnemius muscle. The nerve then enters the lateral compartment of the lower leg as it wraps around the fibular head and runs deeply through the peroneus longus tendon before bifurcating into the deep peroneal nerve and the superficial peroneal nerve. The deep peroneal nerve innervates the anterior compartment of the lower leg and provides sensation to the first web space; the superficial peroneal nerve innervates the lateral compartment of the lower leg and provides sensation to areas of the anterolateral lower leg and dorsal midfoot [4, 5].

The fibular neck is the most frequent area where the peroneal nerve is prone to compression [6]. Some factors may explain this fact: some tethering of the nerve at this point, the increased number of fascicles in this area, the epineural enlargement, and the superficial location of the nerve on the lateral knee location [7].

Compression of the CPN frequently occurs in association with lesions at the neck of the fibula in 54% of all cases; its traumatic etiology represents 40% whereas its tumoral origin stands for only 6% [8]. Spur or tumoral structure formation at the level of the proximal fibula can potentially apply pressure to the surrounding structures especially on the CPN [9].

Osteochondroma (OC), also known as osteocartilaginous exostosis or cartilage-capped exostosis, is a broad (sessile) or narrow (pedunculated) skeletal protrusion comprised of marrow and cortical bone [10]. The cartilaginous cap is the site of growth, which normally diminishes after skeletal maturity. The tumor is presented as a locally benign neoplasm, which favors the metaepiphyseal region of long bones like the distal femur, as well as the proximal tibia or humerus [10]. OC is the most common benign bone tumor accounting for more than a third of all bone tumors [10].

Osteochondromas can develop as a single tumor (solitary osteochondroma (SO)) or as many tumors (multiple osteochondromas (MO)). They are usually asymptomatic but may become painful and cause skeletal deformities, nerve impingement syndromes, malignant transformations, and mass effects.

Clinical manifestations depend on the size and the location of the mass [11]. Non skeletal complications, such as nerve compressions, are extremely rare occurring in less than 1% of all cases [11]. Kim and Kline [12] reported 302 cases of peroneal nerve injuries and described only one patient who presented bilateral peroneal compression by exostosis of the head of the fibula. Only a few cases of entrapment of the peroneal nerve by cartilaginous exostosis have been reported so far [9]. It is assumed that paralysis in our case is secondary to exostosis and provoked by the trauma to the knee.

Motor deficits are more common than sensory nerve lesions, which might be explained by the arrangement of the fascicles inside the common peroneal nerve. The motor fascicles run more medially, whereas the sensorial fascicles are located laterally. The exostosis grows from the bone surface to the periphery, compressing the motor fibers earlier [13].

Electrophysiological tests can help to locate the lesions along the nerve's course and enable us to distinguish the level of the palsy and the extent of sensory and motor impairment. They can also shed light on distinction between entrapment of the peroneal nerve and the sciatic neuropathy or L5 radiculopathy [6].

The first complementary assessment techniques of the common peroneal injury should be plain radiographs and ultrasonography (US). The interest of radiography lies in the proximity of the nerve to the fibular neck and potential for impingement secondary to trauma, tumor, or bony exostosis as shown in our case. US is a noninvasive and cheap screening method. High-resolution ultrasonography may easily evaluate the nerve in its more superficial locations, such as around the fibular head [6]. Also, US can demonstrate the cartilage cap very accurately as a hypoechoic region bounded by bone on its deep surface and muscle/fat superficially [14].

These assessments can direct us towards the etiological diagnosis as what was observed in our case. Otherwise, other second-line examinations may be performed, including magnetic resonance imaging (MRI) and CT scan. MRI provides a better assessment of regional anatomical relationships. It elucidates the position and the composition of a suspected tissue which can compress the common peroneal nerve during its course. MRI is also found to be the best imaging modality to estimate the cartilage thickness (and thus assessing for malignant transformation) in OC [14, 15]. CT scans provide better analysis of bone abnormalities and the cartilage cap [14, 15]. Since the diagnosis of osteochondroma was found to be obvious, MRI and CT were not performed in our patient.

Presence of an exostosis is, in itself, an insufficient reason for its surgical excision, especially in isolated cases. Surgical removal is indicated if the tumor causes pain or functional incapacity, limitation of joint range of motion, or neurovascular compression such as in our case in the fibular head region [15, 16]. Surgery for an osteochondroma causing peroneal nerve entrapment should be performed within three months; otherwise, the surgical success rate decreases and nerve damage becomes irreversible [9, 16].

## 4. Conclusion

Drop foot is an emergency that should rapidly engage the clinician to identify the underlying etiology. In this regard, compressive exostosis constitutes one of the seldom causes of CPN palsy. In our patient, surgical excision was associated with favorable outcome and prevented irreversible functional impairments.
